# ALTERATIONS IN TARGETED METABOLOMICS PRIOR TO UNINTENTIONAL WEIGHT LOSS IN OLDER ADULTS

**DOI:** 10.1093/geroni/igz038.3415

**Published:** 2019-11-08

**Authors:** Mary Elizabeth Baugh, Olga R Ilkayeva, James R Bain, Michael J Muehlbauer, Megan M Marron, Anne B Newman, Samaneh Farsijani, Stephen Kritchevsky

**Affiliations:** 1 Wake Forest School of Medicine, Winston-Salem, North Carolina, United States; 2 Sarah W. Stedman Nutrition and Metabolism Center, Duke Molecular Physiology Institute, Durham, North Carolina, United States; 3 University of Pittsburgh, Pittsburgh, Pennsylvania, United States

## Abstract

In older adults, unintentional weight loss (UWL) is associated with poor outcomes, but its pathophysiology remains poorly understood. We sought to identify potential biomarkers of UWL using targeted metabolomics, including 8 conventional metabolites, 45 acylcarnitines, and 15 amino acids. We identified individuals from the Cardiovascular Health Study All Stars with UWL (n=40) or weight stability (WS; n=40) from Years 9 to 11. Participants had WS through Year 8. UWL was defined as experiencing >6% weight loss from Years 9 to 11 and self-reporting that loss as unintentional. Mean plasma metabolite concentrations measured in Year 9 were compared between individuals with UWL or WS between Years 9 and 11. The strongest signals in metabolomic differences between individuals going on to experience UWL versus WS were observed among the branched-chain amino acids, valine (236.54 ± 54.43 vs. 215.79 ± 32.69 μM, 95%CI: -40.81, -0.70) and isoleucine/leucine (159.09 ± 36.53 vs. 142.75 ± 23.78 μM, 95%CI:-30.10, -2.59); lactate (1.23 ± 0.44 vs. 1.00 ± 0.57 μM, 95%CI: -0.45, -0.001); histidine (35.69 ± 5.33 vs. 38.62 ± 4.86 μM, 95%CI: 0.65, 5.20); the medium-chain acylcarnitine octenoyl carnitine (C8:1) (0.23 ± 0.10 vs. 0.29 ± 0.14 μM, 95%CI: 0.01, 0.12); and long-chain acylcarnitine myristoyl carnitine (C14) (0.04 ± 0.01 vs. 0.03 ± 0.01 μM, 95%CI: -0.01, -0.002). These findings suggest altered branched-chain amino acid and fatty acid metabolism and increased oxidative stress and inflammation may be evident before individuals undergo UWL. Further investigation of these pathways may reveal novel preventive or treatment strategies for UWL.

